# Design and Characterization of Zeolite/Serpentine Nanocomposite Photocatalyst for Solar Hydrogen Generation

**DOI:** 10.3390/ma15186325

**Published:** 2022-09-12

**Authors:** Abeer S. Altowyan, Mohamed Shaban, Zeinab M. Faidey, Khaled Abdelkarem, Mawaheb Al-Dossari, N. S. Abd El-Gawaad, Mohamed G. M. Kordy

**Affiliations:** 1 Department of Physics, College of Science, Princess Nourah bint Abdulrahman University, P.O. Box 84428, Riyadh 11671, Saudi Arabia; 2 Nanophotonics and Applications Lab, Physics Department, Faculty of Science, Beni-Suef University, Beni-Suef 62514, Egypt; 3 Department of Physics, Faculty of Science, Islamic University of Madinah, P.O. Box 170, Al Madinah Al Monawara 42351, Saudi Arabia; 4 Geology Department, Faculty of Science, Beni-Suef University, Beni-Suef 62514, Egypt; 5 Department of Physics, Faculty of Science, King Khalid University, Abha 62529, Saudi Arabia; 6 Faculty of Science, King Khalid University, Mohayel Asser, Abha 61421, Saudi Arabia; 7 Biochemistry Department, Faculty of Science, Beni-Suef University, Beni-Suef 62521, Egypt

**Keywords:** photoelectrochemical hydrogen generation, zeolite, serpentine, nanocomposites, hydrothermal approach

## Abstract

In this work, a low-cost, high-yield hydrothermal treatment was used to produce nanozeolite (Zeo), nanoserpentine (Serp), and Zeo/Serp nanocomposites with weight ratios of 1:1 and 2:1. At 250 °C for six hours, the hydrothermal treatment was conducted. Various methods are used to explore the morphologies, structures, compositions, and optical characteristics of the generated nanostructures. The morphological study revealed structures made of nanofibers, nanorods, and hybrid nanofibril/nanorods. The structural study showed clinoptilolite monoclinic zeolite and antigorite monoclinic serpentine with traces of talcum mineral and carbonates. As a novel photoelectrochemical catalyst, the performance of the Zeo/Serp (2:1) composite was evaluated for solar hydrogen generation from water splitting relative to its constituents. At −1 V, the Zeo/Serp (2:1) composite produced a maximum current density of 8.44 mA/g versus 7.01, 6.74, and 6.6 mA/g for hydrothermally treated Zeo/Serp (1:1), Zeo, and Serp, respectively. The Zeo/Serp (2:1) photocatalysts had a solar-to-hydrogen conversion efficiency (STH) of 6.5% and an estimated hydrogen output rate of 14.43 mmole/h.g. Consequently, the current research paved the way for low-cost photoelectrochemical catalytic material for efficient solar hydrogen production by water splitting.

## 1. Introduction

Fossil fuel burning is the major source of CO_x_ emissions (CO_2_ and CO) in atmospheric air which causes global warming. The resulting air pollution can have catastrophic effects on humans and animals alike. Hydrogen fuel is a carbon-free, renewable, and environmentally friendly source of energy that can be used as an ideal alternative to fossil fuels. Therefore, the development of effective techniques for massive production of hydrogen fuel at a reasonable cost is an important research area. The photoelectrochemical (PEC) hydrogen production utilizing naturally occurring metal oxide-based catalysts is a promising technique to meet these requirements [1]. In the PEC process, the photocatalyst produces an electron/hole pair after absorbing a photon, which is then isolated, transported, and contributed to the cathodic hydrogen evolution/anodic oxygen evolution reactions at applied voltage [2,3]. Therefore, one of the current key objectives is to develop affordable, efficient, and large-scale photoelectrochemical catalysts from natural resources.

Zeolites are classified as crystalline inorganic materials made of SiO_4_ and Al_2_O_3_ tetrahedra with a structure packed with ions and water molecules and exhibiting significant mobility [4]. Due to their great qualities including their semiconducting nature, high ion-exchange capacity, huge surface area, high chemical inertness, photochemical stability, adjustable hydrophobicity/hydrophilicity, and zero toxicity, zeolites have previously been used as stable carriers for various applications, such as their aid in the creation of metal nanoparticles [4]. Due to the superior adsorption on their surfaces for a variety of functions, zeolites have become the focus of research in recent years [5,6]. Since the exchangeable cation has been recognized in the molecular structure of zeolites, both their ion exchange and adsorption capacities have been explored [7]. Natural zeolites have a high selectivity for heavy metal cations such as Pb^2+^, Cu^2+^, Ni^2+^, and Cd^2+^ and non-metal cations such as ammonium ions [8,9]. Therefore, the researchers focused their studies on using zeolites for wastewater treatment and the removal of heavy metals from polluted water. To expand the uses of zeolites, scientists modified the natural zeolite and also created synthetic zeolites. Zeolites were modified to remove Ca^2+^ and Mg^2+^ ions, which are the principal causes of hardness in aqueous solutions and raw groundwater. This was done by leveraging their ion exchange and adsorption characteristics [5]. Organic dyes such as Congo red were photodegraded and adsorbed using natural and synthesized zeolites [6,10].

Mostafa and Ehab prepared zeolite nanostructures by the hydrothermal treatments of Si and various Al sources for the adsorption and photocatalytic degradation of MB molecules from aqueous media [11]. You and Chen prepared TiO_2_/zeolite nanocomposites using the sol-gel technique for the effective delocalization of Rhodamine B (RhB) by the photogenerated electrons [12]. Lei et al. studied the superior photocatalytic decolorization of MB solution using CuO/TiO_2_/zeolite nanocomposite with high stability and reusability [13]. Farhana et al. prepared Fe_2_O_3_-supported zeolite for photo decolorization of methyl orange (MO) [14]. Shaban et al. designed a bentonite/zeolite-NaP nanoporous composite for the photodegradation of MB and Congo red dyes [15]. Abukhadra et al. synthesized nano photocatalyst of zeolite/polyaniline/nickel oxide to effectively photodegrade the Safranin-T dye using sunlight [16]. WO_3_ nanoparticles have also been loaded to zeolite for effective photodegradation of RhB using solar light [17]. The acceleration of RhB’s degradation rate was due to the efficient separation of the electrons/holes and the availability of active sites to adsorb RhB molecules. Moreover, zeolite was used as a support for semiconductor-based PEC catalysts to enhance the hydrogen production rate. The ZnCo/CdS/zeolite heterostructure was optimized by Jia-Hui et al. to achieve photocatalytic hydrogen activity of 59 times greater than that of pristine CdS, which contributed to zeolite’s role in improving the separation and transportation capacity of photo-generated charge carriers [18]. Yue and Khan reported the formation of vacant sites on the zeolite surface due to the exchange of ions in titano-zeolites, which assists the hydrogen photoproduction [19]. Furthermore, Pt/zeolite and Cu/zeolite were prepared and applied for the hydrogen [20,21].

Serpentines, on the other hand, are typically found in serpentinite rocks, which are the source of Mg^2+^ and fibrous silica and are used as decorative stones due to their magical colors [22]. Serpentine has been divided into three primary polymorphs: antigorite, lizardite, and chrysotile due to the comparatively varied distribution of the proportions of hydrated magnesium, silicates, and metal ions such as Fe^2+^, Fe^3+^, Al^3+^, Ni^2+^, Mn^2+^, and Zn^2+^ in serpentine [22]. Shaban et al. used naturally existing cracked serpentine to successfully remove three typical pollutants from wastewater, including methylene blue, Congo red, and Cr^5+^ cations [23]. Upon examination, it was shown that serpentine had 40.2% MgO, 46.3% SiO_2_, 7.2% Fe_2_O_3_, 2.48% Al_2_O_3_, and 1.5% Cr_2_O_3_ [23]. Likewise, there are trace oxides from CaO (1.13%), TiO_2_ (0.41%), Na_2_O (0.46%), and K_2_O (0.34%) [23]. They showed the good adsorption ability of serpentine for the previously mentioned contaminants [23]. To remove Cr^6+^ from wastewater, Farahat et al. simply modified serpentines by employing them as a host adsorbent of the banded ironstone formations [24].

Zeolite, a naturally occurring, microporous mineral, was chosen for this study to serve as a support and host for serpentine in the creation of our novel nanocomposite with increased surface area. Zeolite’s porous structure allows it to absorb more light because the possibility of light absorption increases with increasing porosity due to light scattering inside these pores. Additionally, it might have more active sites and unique nanotextures, enabling the incorporation and organization of photoactive materials that can be used as photocatalysts to boost their efficiency. In addition, despite its many uses, low cost, abundance, and outstanding semiconducting properties, there has, as far as we are aware, been no research on the use of serpentine, serpentine/zeolite composites, or their nanostructures for solar hydrogen production by water splitting. In this study, we synthesized nanostructured serpentine, zeolite, and zeolite/serpentine nanocomposites with various ratios using the hydrothermal treatment. The manufactured samples are evaluated using a variety of instruments and used for the first time to produce hydrogen using the PEC technique. Both the quantity of hydrogen generated and the efficiency at which solar light converts to hydrogen are estimated.

## 2. Experimental Details

### 2.1. Materials

Representative natural clinoptilolite zeolite and serpentine samples were acquired by Al Nasr company for mining, Egypt. According to Masoud et al., the two primary forms of Egyptian serpentine can be found as antigorite or chrysotile in different ratios [25].

### 2.2. Method

#### 2.2.1. Synthesis of the Hydrothermal Zeolite and Pure Serpentine Samples

Separately, clinoptilolite zeolite and serpentine were milled for 8 h at a constant speed of 1200 rpm. Each sample weighed 5 g, which was then dissolved in 80 mL of 2 M KOH and sonicated for 30 min. They were enclosed within the Teflon-lined autoclave made of stainless steel. They were warmed for 6 h at a temperature of 250 ± 2 °C. To remove excess KOH, the precipitates were then rinsed with distilled water separately numerous times. Finally, samples of zeolite and serpentine were taken from the precipitates produced and dried in an oven at 50 ± 2 °C.

#### 2.2.2. Synthesis of Zeo/Serp (2:1) Nanocomposites

The same hydrothermal techniques were used to create Zeo/Serp nanocomposites in the ratios of (1:1) and (2:1). To create the Zeo/Serp (2:1) composite, 3.33 g of natural clinoptilolite zeolite and 1.67 g of natural serpentine were combined. For Zeo/Serp (1:1) composite, 2.5 g of clinoptilolite zeolite and 2.5 g of serpentine were mixed. The mixtures were sonicated for 30 min after being dissolved in 80 mL of 2 M KOH. Zeo/Serp composites were also produced in a stainless-steel autoclave coated with Teflon. At 250 ± 2 °C, they warmed up for six hours. To remove excess KOH, the precipitates were then rinsed with distilled water separately numerous times. Finally, the Zeo/Serp (2:1) and Zeo/Serp (1:1) composites precipitate samples were dried in an oven at 50 ± 2 °C.

### 2.3. Characterization of the Hydrothermally Prepared Samples

Characteristic X-ray diffraction patterns (XRD) of Cu K-alpha radiations in the 2theta range from 5° to 80° were observed using an APD-3720 diffractometer (Philips X’Pert Pro MRD) (Malvern, UK). Operating at 20 mA, 40 kV, and a scanning speed of 5°/min, it was able to identify the crystallographic features of the original zeolite and serpentine samples as well as the Zeo/Serp (2:1) composite sample. The nanomorphologies of the fabricated samples were examined utilizing Field Emission Scanning Electron Microscopy (FESEM, Sigma 500 VP, Zeiss, Oberkochen, Germany). The chemical compositions of the as-prepared samples were investigated using EDX integrated SEM detector (AMETEK, Inc.) (Berwyn, PA, USA). The Fourier transformer infrared (FTIR) analysis was conducted on the prepared samples to detect the functional groups present in our samples. The samples were mixed with standard KBr for FTIR measurements from 4000 to 400 cm^−1^ with a resolution of 4 cm^−1^ (FTIR-8400 S Shimadzu, Milton Keynes, UK). The absorbance spectra were measured using UV/Vis/IR spectrophotometer (Lambda 950, Perkin Elmer, Singapore).

## 3. Results and Discussion

### 3.1. The XRD Study and Optical Absorption

**Figure 1:**
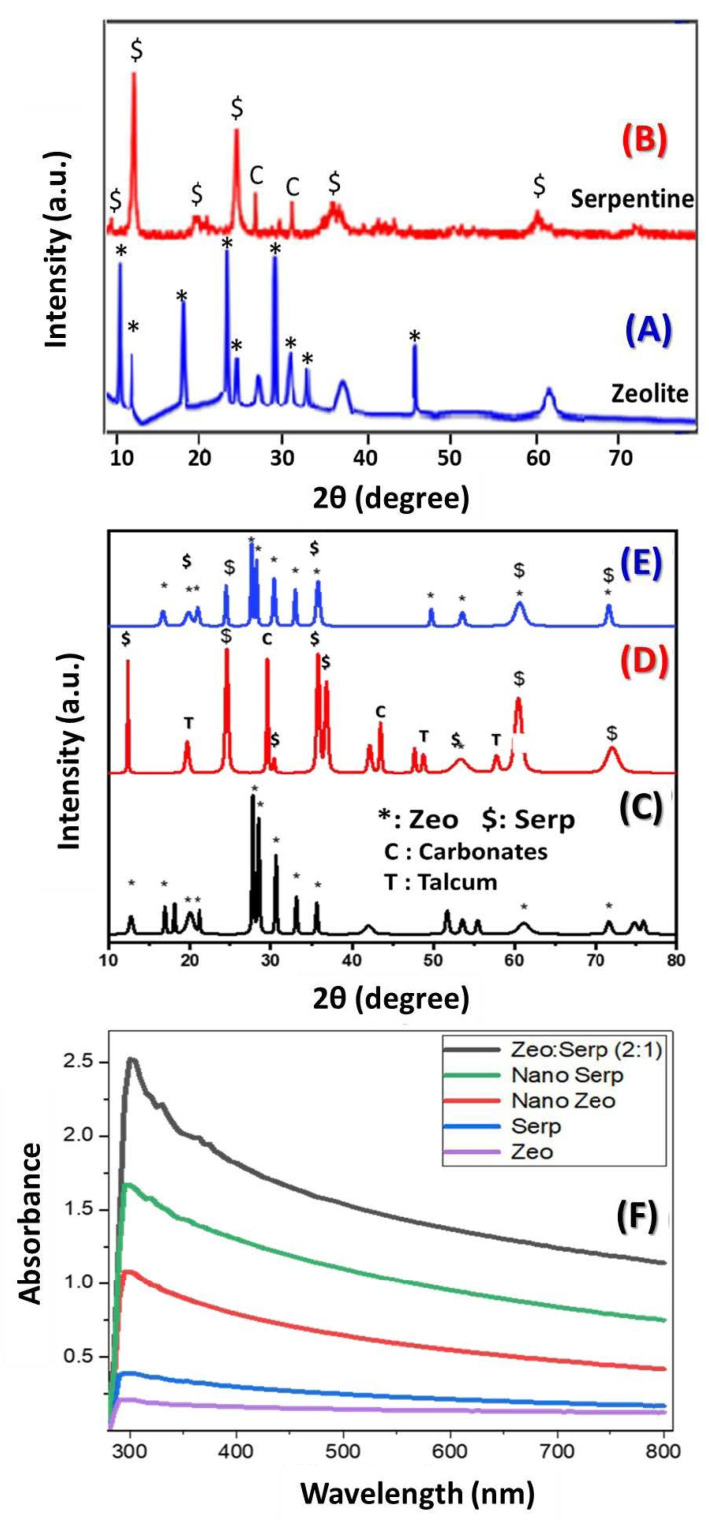
XRD patterns of zeolite (**A**) and serpentine (**B**) after ball milling; XRD patterns of zeolite (**C**), serpentine (**D**), and Zeo/Serp (2:1) composite (**E**) after hydrothermal; and (**F**) optical absorbance spectra of the different samples.

The crystallinity of zeolite, serpentine, and Zeo/Serp (2:1) composite was studied using XRD in the 2θ range from 10° to 80°. Figure 1A displays XRD charts of zeolite and serpentine after ball milling. The characteristic clinoptilolite peaks are seen at different 2θ positions as indicated in the chart of the zeolite. Antigorite predominates the sample in the serpentine chart in Figure 1B and is evident with its clearly strong peaks at positions of 12.3°, 24.5°, and 35.9°. The XRD pattern of zeolite after hydrothermal treatment, Figure 1C, exhibited the distinctive peaks of the clinoptilolite in the zeolite sample that was identified by JCPDS No. 98-010-0096 [26]. The XRD peaks of zeolite change after hydrothermal treatment, becoming sharper and more intense, signifying an improvement in crystallinity.

The molecular structure of the sample was (Na, K, Ca)_2-3_Al_3_(Al, Si)_2_Si_13_O_36_·12H_2_O, which is in good agreement with the stated data in references [27,28]. The XRD pattern of serpentine, Figure 1D, aligns well with that of antigorite (JCPDS card No. 02-0095) with minor existence of talcum mineral (JCPDS card No. 73-0147) and carbonates (JCPDS card No. 00-005-0586) [24]. The hydrothermally created Zeo/Serp (2:1) composite’s XRD investigation, Figure 1E, however, demonstrated the presence of all mineral phases in both zeolite and serpentine ores. The XRD patterns clearly show that the primary component of the Zeo/Serp (2:1) composite was zeolite peaks, while the minor component was serpentine. The presence of the sharp peaks indicated the hydrothermally treated samples to be polycrystalline, which is consistent with the FESEM observations. Due to their low relative intensities and low ratios of Serp to Zeo in the optimized composite, some serpentine-related peaks vanish in the composite material.

Figure 1F shows the optical absorbance spectra of the different samples from 280 to 800 nm. As shown in the figure, the absorbance of the clinoptilolite zeolite and serpentine samples are highly improved after the hydrothermal treatment. The highest absorbance was observed for the Zeo/Serp (2:1) nanocomposite sample. The absorbance peak is shifted from ~292 nm for the zeolite sample to ~305 nm for the nanocomposite. In addition, this peak becomes broader and more intense. The strong absorbance of the nanocomposite makes it more suitable for solar energy applications including photocatalytic dye removal and photoelectrochemical water splitting.

### 3.2. FTIR Study

**Figure 2:**
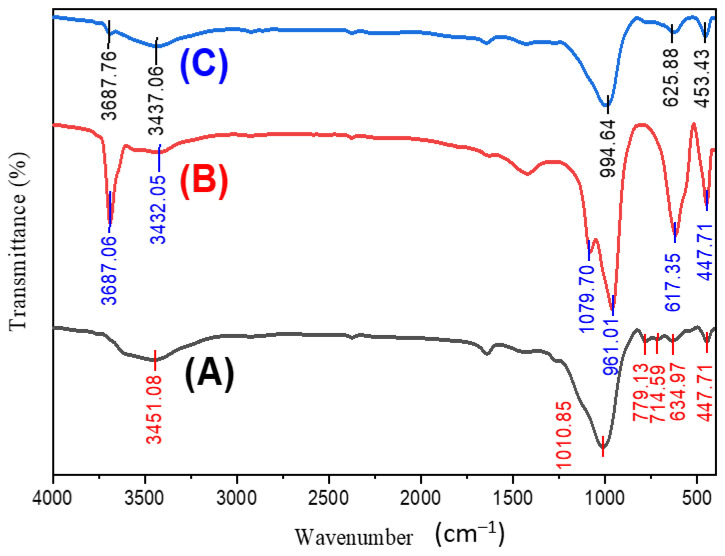
FTIR charts of hydrothermally prepared zeolite (**A**), serpentine (**B**), and Zeo/Serp (2:1) nanocomposite (**C**).

The FTIR spectra of our hydrothermally produced zeolite, serpentine, and Zeo/Serp (2:1) nanocomposite are shown in Figure 2.

The inner OH stretching hydroxyl group that is found in the zeolite and serpentine samples in the aluminosilicates’ structures is shown by the broad band of the hydroxide groups that is detected in the three samples at 3451.08, 3432.05, and 3437.06 cm^−1^, respectively. Serpentine has bands at 961.01 cm^−1^ and 1079.70 cm^−1^, while in the case of zeolite, the band at 1010.85 cm^−1^ may correlate to the Si-O vibration mode. For Zeo/Serp (2:1), these bands were shifted to 994.64 cm^−1^. When Hamd et al. analyzed zeolite composites with algae, the band at 1029 cm^−1^ shifted into 1039 cm^−1^; this may have been due to the produced sample’s asymmetric stretching mode of Si–O–Al or Si–O–Si [6]. Zeolite showed four bands between 400 and 800 cm^−1^, however, Zeo/Serp (2:1) only showed two bands in this range. The other bands’ clarity was diminished by the two peaks’ extraordinary sharpness. These two bands, which are located in the 400–800 cm^−1^ range, are related to the Si–O–Si and Si–O–Al bending modes of the metal oxides present in our samples [29,30].

### 3.3. SEM Morphologies

**Figure 3:**
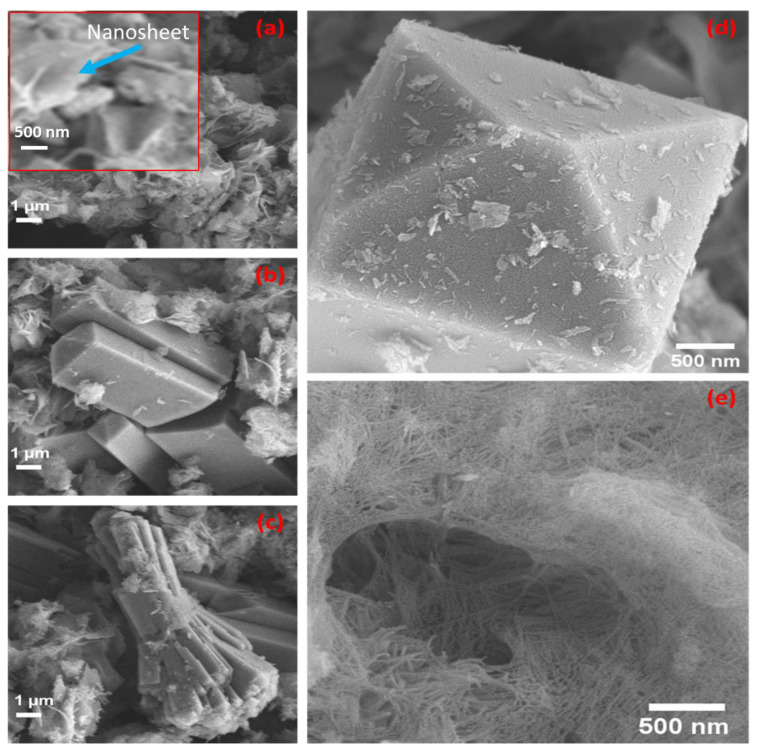
(**a**–**e**) FESEM images of a zeolite sample after undergoing hydrothermal treatment at 250 °C for 6 h showed various morphological features. The inset of (**a**) shows a magnified SEM image of the nanosheet.

Figure 3a–c illustrate, respectively, nanosheets and polygonal regular crystalline particles mixed with nanofibril structures. Zeolite’s textured surface patterns gave a large surface area with outstanding catalytic activity [31]. Nanosheets that were evenly dispersed across the sample matrix are shown in Figure 3a. The polygonal structures have different lengths as shown in Figure 3b–d. The larger constructions of them were not agglomerated as in Figure 3b,d but rather existed singly. The nanofibers, in Figure 3e, were also fully distributed like nanosheets in all sample matrices of the hydrothermally prepared zeolite. A unique structure at this nanoscale is seen in Figure 3e, which is composed of nanofibers or nanofibrils that are bundled together like trawl nets.

**Figure 4:**
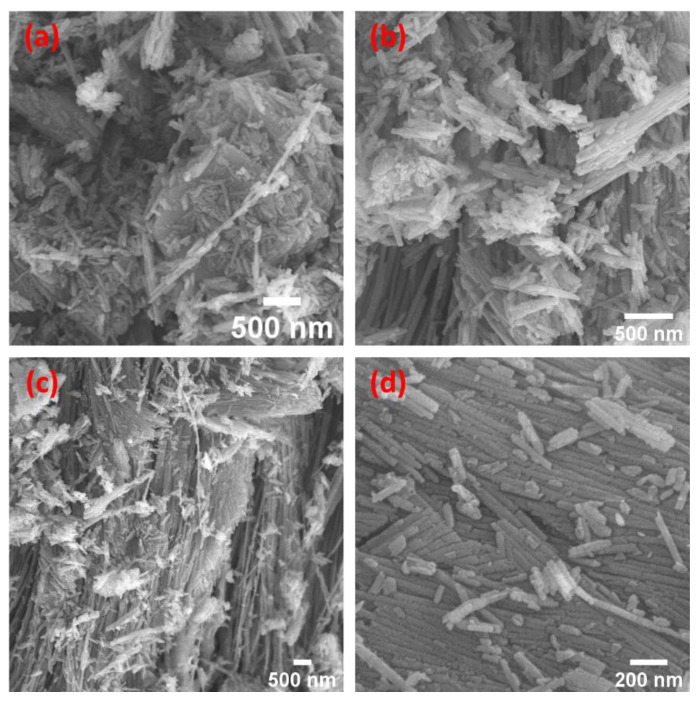
(**a**–**d**) FESEM images at different areas showed nanofibrils with different lengths in the serpentine sample that was hydrothermally treated at 250 °C for 6 h.

In Figure 4a–d, agglomerated and non-agglomerated nanorods were seen in the SEM images of the serpentine sample. As seen in Figure 4c, the bundles of the agglomerated nanorods were queued and laterally oriented. Figure 4 shows how those that were not aggregated had short lengths, existed by themselves, and were scattered around like chalk on a wooden table. These new nanostructures were discovered in our samples after they were hydrothermally produced at 250 °C.

**Figure 5:**
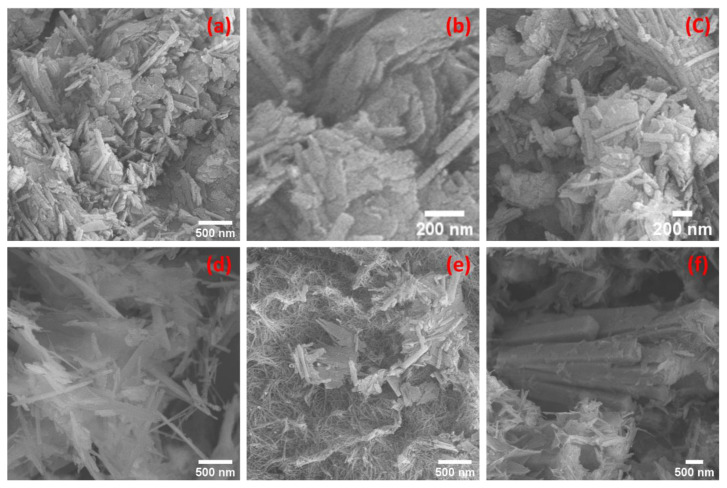
(**a**–**f**) FESEM images at different locations showed the mixed structures of zeolite and serpentine in the Zeo/Serp (2:1) composite sample after hydrothermal treatment at 250 °C for 6 h.

All of the features that were present in the samples discussed before could be seen in the Zeo/Serp (2:1) composite in Figure 5a–f.

As a result, the majority of the structures that were observed belonged to the zeolite sample given that it was present in larger quantities than the serpentine sample. The considerable surface area that these structures offer makes them suitable for use in catalysis. Figure 5b showed accumulated amounts of plates. In addition to these structures, Figure 5d marks the first appearance of the needle structures. This could be because serpentine nanorods that were mixed with zeolite underwent hydrothermal changes. To remove organic dyes from wastewater by adsorption or photocatalysis and to remove Mg^2+^ and Ca^2+^ via adsorption, the rough surfaces of microstructures of natural or modified materials, such as our samples, were utilized as nano-catalysts [5,6].

### 3.4. EDAX Study

**Figure 6:**
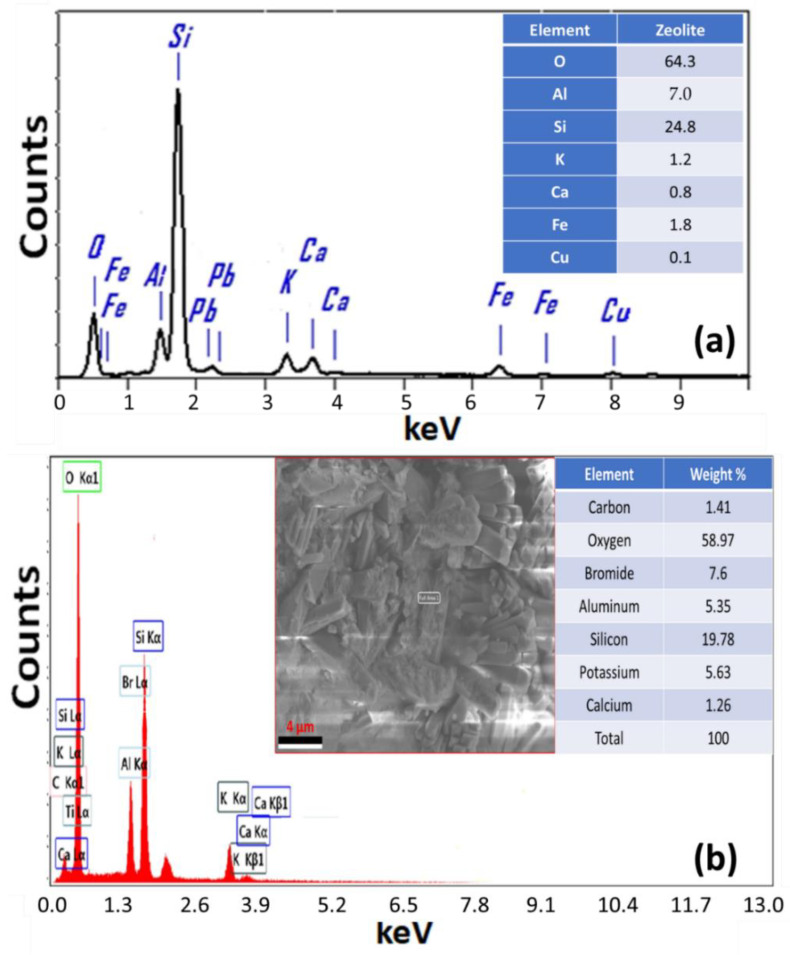
EDAX spectrum of hydrothermally prepared (**a**) zeolite and (**b**) Zeo/Serp (2:1) composite sample.

Figure 6a shows the EDAX chart of the zeolite. This spectrum showed signals of O, Si, Al, Fe, K, Ca, and Cu with weight% of 64.3%, 24.8%, 7.0%, 1.8%, 1.2%, 0.8%, and 0.1%, respectively. The Zeo/Serp (2:1) composite was subjected to EDAX analysis to detect the elemental composition ratios as shown in Figure 6b. In addition to O, Si, Br, K, and Al, which are essential components of the Zeo/Serp (2:1) composite crystal structure, the C and Ca impurities were also discovered in trace amounts. The weight% of the detected signals was 58.97%(Si), 19.78%(Si), 7.60%(Br), 5.63(K), 5.35%(Al), 1.41%(C), and 1.26%(Ca).

### 3.5. Photoelectrochemical (PEC) Water Splitting Measurements

#### 3.5.1. Photoelectrochemical Stability and Behavior

**Figure 7:**
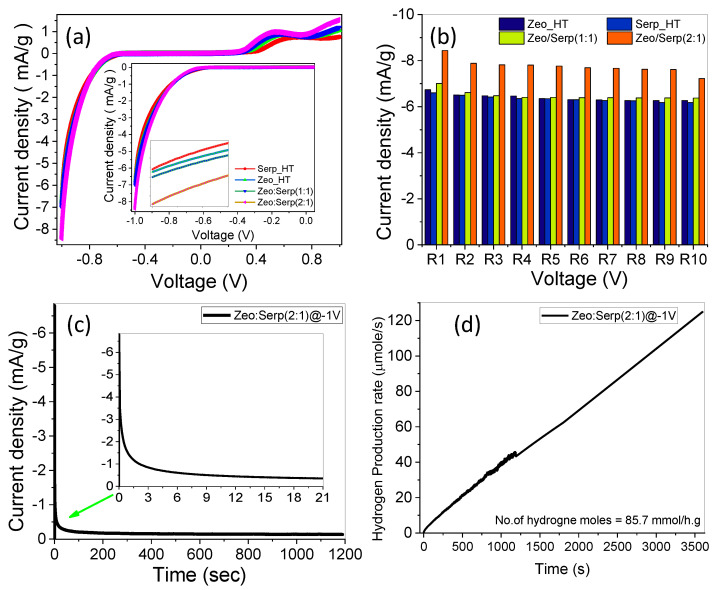
Variation of current density vs. the applied voltage for (**a**) all photocatalysts under standard white light luminance; (**b**) Zeo/Serp (2:1) composite photocatalyst for repeated runs under white lighting conditions; (**c**) current density vs. exposure time for Zeo/Serp (2:1) composite photocatalyst @-1V, and (**d**) number of hydrogen moles versus production time.

The photoelectrochemical properties of the clinoptilolite zeolite and serpentine photocatalysts were measured under a standard white illuminance (AM 1.5 G, 100 mW/cm^2^) and evaluated with the use of a 400 W mercury xenon light source (Newport, MODEL: 66926-500HX-R07, Newport, UK). The OrigaFlex potentiostat (OGFEIS linked to an OGF500 Pack, France) was used to obtain all measurements. With graphite acting as the working electrode and a Pt electrode functioning as the auxiliary electrode, we used 0.05 g of our photocatalyst dispersed in a 0.5 mol/L solution of sodium sulfite heptahydrate (Na_2_SO_3_, 7H_2_O) as the electrolyte, i.e., the working electrode and the auxiliary electrode were dipped in a solution of 0.05 g of our photocatalyst in a 0.5 mol/L (Na_2_SO_3,_ 7H_2_O) electrolyte. The variation of the current density under white light vs. the applied voltage in the range of −1 V to +1 V is shown in Figure 7a. The photo-current density was greatly increased vs. the negative bias voltage and slightly increased vs. the positive bias voltage when all the photocatalysts were used and standard white illuminance was used. The photoelectrochemical current density rose for all electrodes when the negative applied voltage was raised, as predicted by an improvement in the tunnelling action of the photogenerated carriers. As seen in Figure 7a, photo-current density increased when the ratio of zeolite was greater than the serpentine ratio in the mixture. This might be owing to the expansion of the optical bandgap into the visible light range as a result of the increasing zeolite ratio compared to the serpentine ratio, the photoelectrochemical procedure is made possible by these facilities. At −1 V, the Zeo/Serp (2:1) composite produced a maximum current density of 8.44 mA/g. When compared to other photocatalysts, the maximum currents were 7.01, 6.74, and 6.6 mA/gm for Zeo/Serp (1:1), Zeo_HT, and Serp_HT, respectively. This demonstrates that despite the serpentine CB being only slightly more negative, adding zeolite causes a higher rise in current density than adding serpentine because the zeolite CB is substantially more negative. It enhances the driving forces of the migration of holes relative to electron migration. This causes a decrease in e^−^/h^+^ recombination rates and can thus result in efficient charge carrier separation. Hence, the effective e^−^/h^+^ separation occurs over robust interfacial interactions in Serp/Zeo structure, which reduces the e^−^/h^+^ recombination and improves the photo electrocatalytic performance. In addition, the trapping of electrons by the distributed Serp nanorods on the surface of the zeolite can improve the photocatalytic performance of Zeo/Serp [32]. Additionally, the higher ratio of Zeo relative to Serp allows Serp to distribute over the surface of Zeo without blocking its microporous structure, which allows for higher surface area and more light absorption due to the light scattering inside this structure. Moreover, Zeo/Serp (2:1) might have more active sites than Zeo/Serp (1:1), boosting its photocatalytic efficiency.

The performance of the Zeo/Serp (2:1) physical mixture is tested and presented as Supplementary Materials (Figure S1) in order to distinguish between the composite and one physical mixture. The current density for the physical mixture was 6.67 mA/g while it was 8.44 mA/g for the nanocomposite with the same ratio. This amplified photo-catalytic efficacy of the improved composite is evidently demonstrated.

To investigate the photocatalyst’s reproducibility, the photo-current density vs. bias voltage curve for Zeo/Serp (2:1) composite photocatalyst was done in the dark environment and under standard white illumination for 10 runs as shown in Figure 7b. At −1 V, the current density for the Zeo/Serp (2:1) composite photocatalyst was (6.28 mA/gm) in dark compared to the white light current density (8.44 mA/gm) in the first run, indicating that the photoelectrochemical water splitting mechanism is more efficient in light, also after 10 consecutive cycles at −1 V, the photocurrent density decreased by 14.42%, from 8.44 mA/gm to 7.22 mA/gm, showing excellent reproducibility and stability for the optimized photocatalyst (Zeo/Serp (2:1)) in white light at RT. The stability of the Zeo/Serp (2:1) composite photocatalyst for hydrogen production in 0.5 M Na_2_SO_3_, 7H_2_O under standard white light illumination and an applied voltage of −1 V was investigated for an extended period (1200 s). Figure 7c shows the fluctuation of current density vs. time. The current density dropped significantly within the first 180 s, reaching about 0.176 mA/gm. Despite the early decline in photocurrent density, a slight drop in current density was observed for times > 180 s before approaching a constant value of about 0.14 mA/gm. This demonstrates that the Zeo/Serp (2:1) composite photocatalyst was extremely stable and has a long lifespan as a photocatalyst in the water-splitting process for hydrogen production.

In Equation (1), Faraday’s equation was used to calculate the number of moles of hydrogen generated by the photoelectrochemical water-splitting process [33].

(1) $$H_{2}\;(moles)=\int_{0}^{t}\frac{Jph}{F}\;dt$$

where *Jph* is photocurrent density, *F* is the faraday constant (96,500 C/mol) and *t* is the period. The ratio of *H*_2_ moles generated as a function of generation time is plotted in Figure 7d using the reported *Jph*-time data in Figure 7c. The estimated hydrogen output rate was 14.43 mmole/h for the optimized Zeo/Serp(2:1) photocatalyst. The solar-to-hydrogen conversion efficiency (*STH*) is the ratio of total hydrogen energy output to total sunlight energy input. Equation (2) can be used to find the total efficiency of the PEC water splitting cell [34]:

*STH* = [(*H*_2_*/S*) × (237 *KJ/mol*)] / [*P_total_* × *A*] (2)

where *H*_2_*/S* is the rate of hydrogen generation per second, *P_total_* represents the total power density of the illuminating light in mW/cm^2^, and *A* is the area of the photoelectrode. The estimated *STH* was *STH* = 6.5% for the Zeo/Seep(2:1) photocatalysts. According to the results, the Zeo/Serp(2:1) composite photocatalyst is an excellent photocatalyst for the hydrogen evolution (*HE*) reaction.

#### 3.5.2. The Effect of Monochromatic Light Illumination and Photoelectrochemical Efficiencies

**Figure 8:**
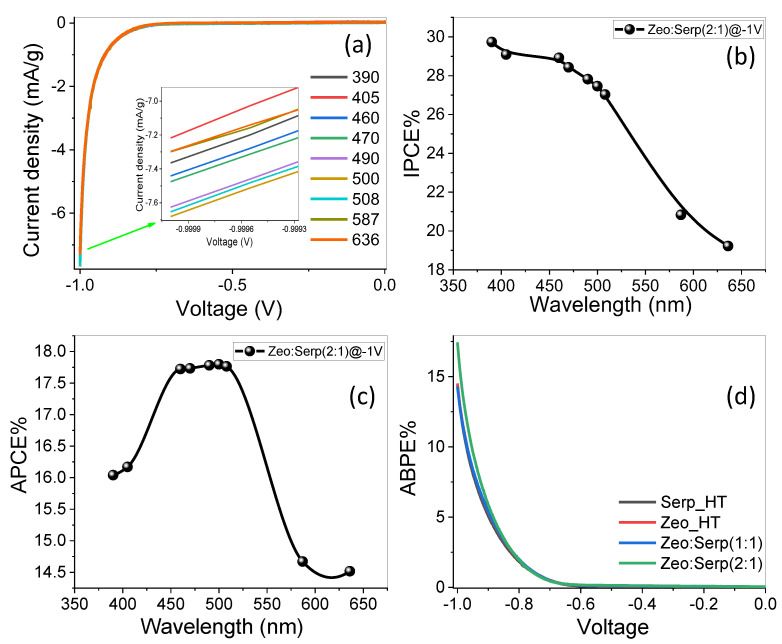
Zeo/Serp(2:1) photocatalyst. (**a**) Variation of Current density vs. the applied voltage under monochromatic luminance, (**b**) IPCE % and (**c**) ABCE % (Wavelength) at −1 V vs. incident wavelength, and (**d**) ABPE % as a function of applied potential.

In 0.5 mol/L (Na_2_SO_3_·7H_2_O) at RT, bandpass filters with wavelengths ranging from 390 to 636 nm were used to investigate the Zeo/Serp(2:1) photocatalyst’s response to monochromatic light and to evaluate its efficiencies in water-splitting process for hydrogen generation. According to Figure 8a, the maximum photocurrent was measured at 500 nm and −1 V and was determined to be *Jph* = 7.68 mA/gm, while the lowest photocurrent was determined to be *Jph* = 7.27 mA/gm at 405 nm. From 390 to 636 nm, the Zeo/Serp(2:1) photocatalyst’s current values are shown to be in a small range. This current density–wavelength range dependency may be related to the Zeo/Serp(2:1) photocatalyst’s absorption behavior at each wavelength, which supports the photocatalytic response of the optimum photocatalyst for the H_2_ production process. In general, this shows that the Zeo/Serp(2:1) photocatalyst is sensitive to a lot of the sun’s light and is good at absorbing a lot of it in the visible range. The Zeo/Serp(2:1) photoelectrode’s improved solar absorption and application to efficient H_2_ generation from H_2_O splitting are further demonstrated by calculating the external quantum efficiency or incident photon-to-current conversion efficiency (*IPCE* %). At various wavelengths of Figure 8a, Equation (3) is used to estimate the *IPCE* % at a fixed voltage of −1 V [33]:

(3) $$IPCE\;\%=1240\;\times \;\frac{Jph}{\left(\lambda .P\right)}\;\times \;100\%$$

where *λ* is the wavelength of the incident photons and *P* is the illuminating light power density of the Xenon lamp as a function of the monochromatic light wavelength. The change in *IPCE %* with wavelength is represented in Figure 8b. At 390 nm, the maximum *IPCE %* of the Zeo/Serp(2:1) photocatalyst is obtained, 29.73%, with an *IPCE %* of 19.22% at 636 nm being the lowest. The influence of optical losses, such as transmission (*T_r_*) or reflection (*R*), was still not considered in the *IPCE %* computations. To compensate for optical losses, the internal quantum efficiency, also known as the absorbed photon-to-current conversion efficiency (*APCE %*), was calculated. The photocurrent generated by each absorbed photon is made up of the number of PEC-generated carriers. *APCE %* is calculated using Equation (4) [35]:

(4) $$APCE(\lambda )=\frac{IPCE\left(\lambda \right)}{A\left(\lambda \right)}=\frac{IPCE\left(\lambda \right)}{1–R–T_{r}}\;$$

where *A(λ)* denotes optical absorption.

The change of *APCE %* as a function of incident wavelength is shown in Figure 8c. This graph shows two significant *APCE %* values: 17.80% around 500 nm and 17.78% around 490 nm, with the lowest value being 14.516% at 636 nm. To calculate the electrode photocatalytic efficiency, remove the extra electrical energy from the system while applying a small external voltage to the *PEC* cell. The applied bias photon-to-current efficiency *(ABPE %)* might be used for this purpose. Equation (5) is used to determine the *ABPE %* values for the designed Zeo/Serp(2:1) [36]:

(5) $$ABPE\;\%=Jph\;\times \;\frac{\left(1.23\;-\;V_{app}\right)}{P}\;\times \;100\%$$

where 1.23 is the standard state reversible potential of H_2_O and *V_app_* is the externally applied voltage. The ratios of *ABPE %* as a function of the applied voltage are shown in Figure 8d. The best value of *ABPE %* for HE reaction were 17.44% for Zeo/Serp(2:1) @-1V, as it was 14.49%, 14.49%, and 14.29% for each of Zeo/Serp(1:1), Zeo_HT, and Serp_HT, respectively. This photocatalyst has excellent *ABPE %* which indicates reduced interfacial transport resistance and enhanced PEC performance. Due to its good performance, the Zeo/Serp(2:1) photocatalyst may be suitable for use in a *PEC* cell. This behavior agrees well with previously reported works focused on the use of Zeo as a support to produce PEC catalysts of a higher rate of hydrogen generation. Due to zeolite’s role in boosting the separation and transportation capacity of photo-generated charge carriers, Jia-Hui et al. developed and refined the ZnCo/CdS/zeolite heterostructure to obtain photocatalytic hydrogen activity that was 59 times more than that of pristine CdS [18]. Yue and Khan assert that the ion exchange in titano-zeolites results in the formation of vacant spots on the zeolite surface that aid in hydrogen photosynthesis [19]. Pt/zeolite and Cu/zeolite were also manufactured and employed for hydrogen [20,21].

In general, the photocatalyst HE reaction on the photocatalyst occurs in multiple processes. First, the semiconductor catalyst absorbs photon energy to produce an electron-hole pair upon photoexcitation. The excited electrons and holes are then separated and migrated to the surface of the photocatalyst. Several electrons and holes will recombine in bulk or on the surface. At the same time, the rest of the charge carriers will reach the catalysts (or catalytic active sites) on the surface where surface redox reactions occur, i.e., HE reaction with photoelectrons and donor oxidation with photo holes [37].

## 4. Conclusions

In this work, nanozeolite (Zeo), nanoserpentine (Serp), and Zeo/Serp nanocomposites with weight ratios of 1:1 and 2:1 were designed employing a low-cost, high-yield hydrothermal process utilizing natural zeolite and serpentine. The hydrothermal treatment lasted 6 h and was carried out at 250 °C. Nanofibers, nanorods, and hybrid nanofibril/nanorod structures were revealed in the morphological investigation. Clinoptilolite monoclinic zeolite and antigorite monoclinic serpentine were found in the structural analysis, along with talcum minerals and carbonates. The Zeo/Serp (2:1) nanocomposite shifts the absorbance peak for the zeolite sample from 292 nm to 305 nm. The effectiveness of the Zeo/Serp (2:1) composite as a new photoelectrochemical catalyst was assessed for solar hydrogen production from water splitting in comparison to its constituents. At −1 V, the Zeo/Serp (2:1) composite generated a maximum current density of 8.44 mA/g, compared to 7.01, 6.74, and 6.6 mA/g, respectively, for hydrothermally treated Zeo/Serp (1:1), zeolite, and serpentine. The current density for the physical mixture Zeo/Serp (2:1) was 6.67 mA/g. The photocurrent density reduced by 14.42% after 10 cycles at −1 V using the optimized Zeo/Serp (2:1) nanocomposite in white light at RT, displaying remarkable consistency and stability. The maximum measured absorbed photon-to-current conversion efficiency (APCE% = 17.80%) value was recorded at 500 nm. Furthermore, the solar-to-hydrogen conversion efficiency (STH) of the Zeo/Serp(2:1) photocatalyst was 6.5%, and the hydrogen generation rate was 14.43 mmole/h.g. As a result of the current research, a low-cost photoelectrochemical catalytic material for effective solar hydrogen synthesis via water splitting is now possible.

## Data Availability

Not applicable.

## Supplementary Materials

The following supporting information can be downloaded at: https://www.mdpi.com/article/10.3390/ma15186325/s1, Figure S1: Variation of photocurrent density vs. the applied voltage for the physical mixture Zeo/Serp (2:1) under white light illumination.
