# Supplementary material for: Design and Characterization of Zeolite/Serpentine Nanocomposite Photocatalyst for Solar Hydrogen Generation
Source: Materials (Basel). 2022 Sep 12;15(18):6325. doi: 10.3390/ma15186325 (PMC9502782; doi:10.3390/ma15186325)
Supplement: Supplementary file 1 [file materials-15-06325-s001.zip › materials-1834971-supplementary.pdf]

Supplementary Materials

Design and Characterization of Zeolite/Serpentine Nanocomposite Photocatalyst for Solar Hydrogen Generation

Abeer S. Altowyan [1], Mohamed Shaban [2,3,*], Zeinab M. Faidey [2,4], Khaled Abdelkarem [2], Mawaheb Al-Dossari [5], N. S. Abd El-Gawaad [6] and Mohamed G. M. Kordy [2,7]

[1] Department of Physics, College of Science, Princess Nourah bint Abdulrahman University, P.O. Box 84428, Riyadh 11671, Saudi Arabia
[2] Nanophotonics and Applications Lab, Physics Department, Faculty of Science, Beni-Suef University, Beni-Suef 62514, Egypt
[3] Department of Physics, Faculty of Science, Islamic University of Madinah, P.O. Box 170, Al Madinah Al Monawara 42351, Saudi Arabia
[4] Geology Department, Faculty of Science, Beni-Suef University, Beni-Suef 62514, Egypt
[5] Department of Physics, Faculty of Science, King Khalid University, Abha 62529, Saudi Arabia
[6] Faculty of Science, King Khalid University, Mohayel Asser, Abha 61421, Saudi Arabia
[7] Biochemistry Department, Faculty of Science, Beni-Suef University, Beni-Suef 62521, Egypt
* Correspondence: mssfadel@aucegypt.edu.

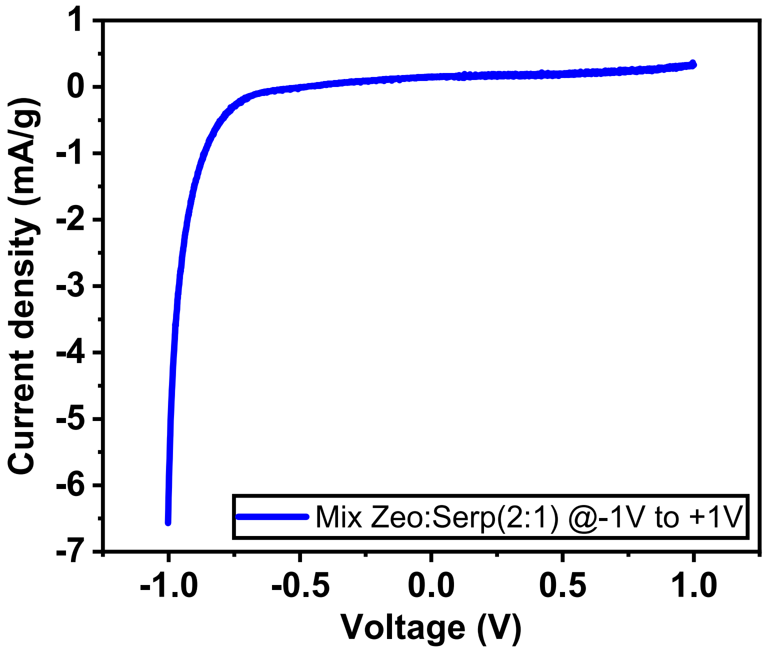

Figure S1. Variation of photocurrent density vs. the applied voltage for the physical mixture Zeo/Serp (2:1) under white light illumination.
